# Supplementary material for: MetaRibo-Seq measures translation in microbiomes
Source: Nat Commun. 2020 Jun 29;11:3268. doi: 10.1038/s41467-020-17081-z (PMC7324362; doi:10.1038/s41467-020-17081-z)
Supplement: Supplementary file 10 — Supplementary Data 7 [file 41467_2020_17081_MOESM10_ESM.zip › File2/Confidence_High_Taxonomy/351167_out.krona.html]

Javascript must be enabled to view this page.

members
magnitude
magnitudeUnassigned
count
unassigned
taxon
rank

351167\_out

6

6
superkingdom
2

976
6
phylum

6
class
200643

order
6
171549

171552
family
6

genus
6
838

species
5
165179

SRS045713\_contig\_number\_21792SRS1041031\_contig\_number\_26335SRS1041140\_contig\_number\_22453SRS144537\_contig\_number\_contig-100\_2392.234174SRS144537\_contig\_number\_contig-100\_11104.277600

species
1
1263102

SRS049959\_contig\_number\_4673
